# Calculation of the Real Corneal Refractive Power after Photorefractive Keratectomy Using Pentacam, When Only the Preoperative Refractive Error is Known

**DOI:** 10.1155/2020/1916369

**Published:** 2020-04-01

**Authors:** Maddalena De Bernardo, Maria Borrelli, Roberto Imparato, Nicola Rosa

**Affiliations:** ^1^Department of Medicine, Surgery and Dentistry “Scuola Medica Salernitana”, University of Salerno, Salerno, Italy; ^2^Department of Ophthalmology, University Hospital Duesseldorf, Duesseldorf, Germany

## Abstract

**Purpose:**

To check if a regression formula, IOLMaster-derived, to calculate the real corneal power after photorefractive keratectomy (PRK), can give reliable results utilizing the Pentacam.

**Methods:**

Pre- and postoperative IOLMaster, Km, and Pentacam K readings were measured. Patients who had myopic PRK were divided into two groups: the first group (108 eyes) was utilized to check which of the preop Pentacam K readings (P-Kpre) better fitted with the preop IOLMaster measurements; in the second group (120 eyes), the real K (Kr), obtained adding the effective treatment to the P-Kpre, were compared with the K readings calculated with the IOLMaster-derived formula (Kc). Moreover, an attempt to find a different formula utilizing the P-Kpre was made.

**Results:**

In group 1, the best correlation was found between IOLMaster Km and Pentacam equivalent K readings (r2 0.9519). In group 2, the comparison between Kr and Pentacam postop Km showed 69 eyes (57%) with differences >0.5 D and 38 eyes (31%) with differences >1 D, (*P* < 0.001). The comparison between Kr and Kc showed 55 eyes (45%) with differences >0.5 D and 22 eyes (18%) with differences >1 D, (*P* < 0.001). Moreover, a regression formula K = EKR − [ETcp + (0.8114 ∗ ETcp − 0.2031)] was obtained in order to calculate the K readings to be used with the Pentacam in the IOL power calculation in case the effective treatment is known.

**Conclusions:**

K calculated with the new formula could be used in patients that underwent refractive corneal surgery in case a Pentacam device is used, pending further studies conducted in clinical practice to establish its accuracy and effectiveness. This study further proves that data obtained from different machines cannot be used interchangeably.

## 1. Introduction

It is well known that, after radial keratotomy, photorefractive keratectomy (PRK), and laser in situ keratomileusis (LASIK), the devices routinely used to measure the corneal power tend to overestimate it [1–4].

For this reason, in case of cataract surgery, the power of the IOL will be underestimated, and the patient will be hyperopic with the need of an IOL exchange or a piggyback lens [3, 4].

Many methods have been described to calculate IOL power after refractive surgery procedures, and they can be mostly divided into two groups depending on the availability of preoperative and postoperative data.

In the literature, some papers suggest a strong correlation between IOLMaster and Pentacam K readings in nonoperated eyes [5, 6, 7].

Among the methods that have been described trying to overcome the IOL calculation problem after refractive surgery [8–25], Rosa et al., in 2004, proposed a regression formula to be used with the IOLMaster when the effective treatment is known, but the preoperative K readings (P-Kpre) are missing [25].

The purpose of this study was to check eventual correlations between postoperative Pentacam and IOLMaster K readings and if the previously described formula could give reliable results utilizing a different device, namely, the Pentacam Scheimpflug camera.

## 2. Materials and Methods

This retrospective clinical study comprised consecutive patients who had PRK for myopia or for myopic astigmatism. The study was conducted in adherence to tenets of the World Medical Association's Declaration of Helsinki. Institutional review board approval was obtained, and informed consent was obtained from all individual participants included in the study. The exclusion criteria, the surgical procedure, and the postoperative treatment were the ones we routinely used in these patients, as described in previous papers [24, 26].

Before and 6 months after PRK, all patients had a complete ophthalmic examination, including automatic K measurements with a rotating Scheimpflug anterior segment imaging (Pentacam, Oculus Optikgerate GmbH, Wetzlar, Germany, version 1.17r20) and an IOLMaster 500 (Zeiss, Jena, Germany, version 5.4.4.00006) evaluation.

The first step was to test before surgery which of the Pentacam parameters better fitted with the IOLMaster measurements.

In the second step, in the patients that reached a six-month follow-up, the real K (Kr) were obtained adding the effective treatment, calculated at the corneal plane, to P-Kpre.

Before and after PRK, the effective treatment was converted at the corneal plane, with the vertex distance correction equation [25].

The Kr were then compared with the postoperative Pentacam equivalent K readings (EKR) at 4.5 mm that is considered to be the optimal zone sample size [27] and with the calculated K readings (Kc) with the previously published formula, found with an IOLMaster.

ΔK = 0.7615 and ΔR − 0.6773 (where ΔR = refractive difference at the corneal plane and ΔK = keratometric difference at 6 months follow-up) [25].

Kc were calculated adding ΔK to the P-Kpre.

Moreover, an attempt was made to try to duplicate the previous work to see if it was possible to find a different formula that could be used with Pentacam data utilizing the P-Kpre [25].

The refraction and the keratometric analyses were performed by 2 independent observers (NR and MDB). The data normality was tested with Kolmogorov–Smirnov test. The correlation between the different K values was assessed by linear regression analysis, and Bland–Altman plots were utilized to analyze the agreement of the measurements provided by the two devices.

## 3. Results

**Figure 1:**
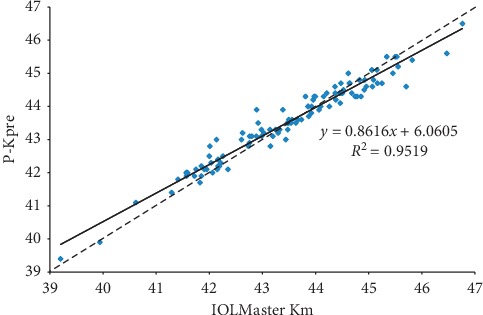
Scatterplot showing correlation between the preoperative IOLMaster mean keratometry (Km) and P-Kpre (preoperative Pentacam equivalent K reading) in diopters (D). Solid = correlation line and dashed = bisector.

The study included for the first step 108 eyes of 54 patients (group 1) (22 women) with a mean age of 32.6 years (SD 8.77) (20–54 years) and a mean preoperative spherical equivalent refraction of −4.7 D (SD 2.35) (−14.5 to −0.5 D). The best correlation between the IOLMaster Km and the Pentacam data was obtained with the P-Kpre (Figure 1).

For the second step, 120 eyes of 60 patients (group 2) (32 women) with a mean age of 33 years (SD 8.9) (19–55 years) and a mean preoperative spherical equivalent refraction of −5 D (SD 2.23) (−14.5 to −0.5 D) were utilized to test the formula.

The postop EKR, Kr, and Kc values are shown in Table 1.

**Figure 2:**
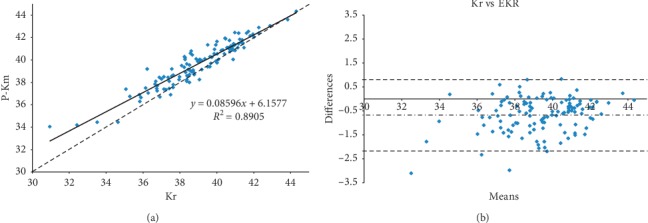
(a) Scatterplot showing correlation between 6 months postoperative Pentacam equivalent K readings (EKR) and real Km (Kr) in diopters (D). Solid = correlation line and dashed = bisector. (b) Bland and Altman plot showing correlation between 6 months postoperative Pentacam equivalent K readings (EKR) and real Km (Kr) in diopters (D), with 95% LoA (range: −1.96 sd to +1.96 sd).

The comparison between Kr and postop EKR (Figures 2(a) and 2(b)) showed a statistically significant difference (*P* < 0.001) with 69 eyes (57%) presenting differences ≥0.5 D and 38 eyes (31%) with differences ≥1 D, leading to roughly a similar error in the IOL power calculation.

**Figure 3:**
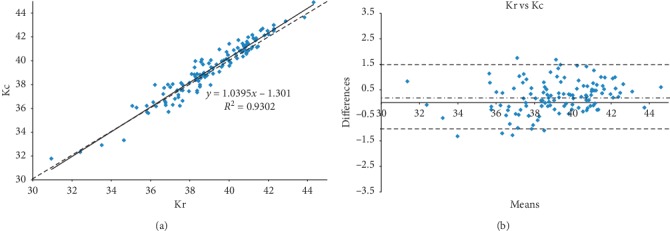
(a) Scatterplot showing correlation between real Km (Kr) and K readings calculated with the previously published formula (Kc) in diopters (D). Solid = correlation line and dashed = bisector. (b) Bland and Altman plot showing correlation between real Km (Kr) and K readings calculated with the previously published formula (Kc) in diopters (D), with 95% LoA (range: −1.96 sd to +1.96 sd).

The comparison between Kr and Kc (Figures 3(a) and 3(b)) showed a statistically significant difference (*P* < 0.001) with 55 eyes (45%) presenting differences ≥0.5 D and 22 eyes (18%) with differences ≥1 D, leading to roughly a similar error in the IOL power calculation.

These results show that both EKR and Kc should not be utilized to calculate the IOL power after refractive surgery.

**Figure 4:**
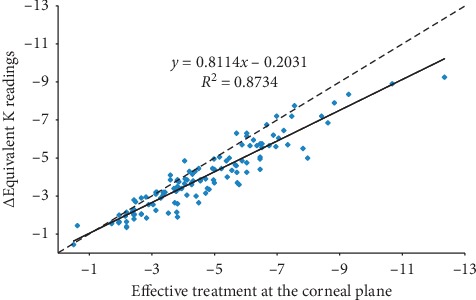
Scatterplot showing correlation between effective treatment at the corneal plane and keratometric difference (Δ) in equivalent K readings at 6 months follow-up, in diopters (D). Solid = correlation line and dashed = bisector.

Moreover, in the attempt made to duplicate the work performed with the IOLMaster (Figure 4), we found the following regression formula to calculate the K readings to be used with the Pentacam in the IOL power calculation, in case the effective treatment is known:

  K = EKR − [ETcp + (0.8114 ∗ ETcp −0.2031)],
  where K = K reading to be used in the IOL power calculation
  EKR = 6 months postoperative Pentacam equivalent K reading
  ETcp = effective treatment at the corneal plane

## 4. Discussion

The influence of refractive surgery on the ocular parameter evaluation, such as intraocular pressure and corneal power, has been widely studied [28].

Three main reasons have been claimed to explain the overestimation of the corneal power after refractive surgery: inaccurate measurement of the anterior corneal curvature by automated and manual keratometry (K) or computerized videokeratography, inaccurate value of the keratometric index resulting from the modified relationship between the anterior and posterior corneal surface, and incorrect estimation of the effective lens position (ELP) resulting from these modifications [8, 9].

In patients that underwent refractive surgery, if the achieved correction and the preoperative K readings are known, it is possible to calculate Kr.

Unfortunately, if the achieved correction is known and the preoperative K readings are unknown, it is not possible to calculate Kr because the difference in K readings does not correspond to the one detected by the machines [1, 3].

Rosa et al., in 2004, studying the reliability of the IOLMaster in measuring corneal power after photorefractive keratectomy, found that this device did not accurately reflect the effective induced refractive changes, particularly in eyes that had high dioptric treatment, and proposed a regression formula which tried to overcome such a problem calculating the real refractive power, when the effective treatment is known, but the preoperative K readings are missing [25].

In the present paper, we demonstrate that this formula cannot be used with Pentacam data, further proving that, in patients that underwent refractive surgery, different devices provide different measurements, and the proposed formulas to overcome the problem of the underestimation of the corneal power after such a surgery cannot be used for all the devices.

In conclusion, K calculated with the new formula, in patients undergoing cataract surgery, could be used in patients that underwent refractive corneal surgery, in case a Pentacam device is used. Further studies conducted in clinical practice will be necessary to establish the accuracy and effectiveness of this new formula.

## Figures and Tables

**Table 1:** Means, standard deviation, and ranges of different K readings in diopters.

	Kr†	Kc‡	EKR§
Mean	39.04	39.27	39.70
SD	2.19	2.35	1.99
Min	30.95	31.78	34.05
Max	44.30	44.91	44.35

†Kr = real Km obtained adding the effective treatment calculated at the corneal plane to the preoperative equivalent K readings. ‡Kc = K readings calculated with the previously published formula^23^. §EKR = Pentacam postoperative equivalent K readings.

## Data Availability

The data used to support the findings of this study are available from the corresponding author upon request.
